# Heat-labile enterotoxin enhances F4-producing enterotoxigenic *E. coli* adhesion to porcine intestinal epithelial cells by upregulating bacterial adhesins and STb enterotoxin

**DOI:** 10.1186/s13567-022-01110-4

**Published:** 2022-10-27

**Authors:** Qiangde Duan, Shengmei Pang, Lili Feng, Jiaqi Liu, Linfen Lv, Baoliang Li, Yuxuan Liang, Guoqiang Zhu

**Affiliations:** 1 College of Veterinary Medicine, Yangzhou University, Yangzhou, 225009 China; 2 Jiangsu Co-innovation Center for Prevention and Control of Important Animal Infectious Diseases and Zoonoses, Yangzhou, 225009 China; 3 Jiangsu Joint Laboratory for International Cooperation in Prevention and Control of Important Animal Infectious Diseases and Zoonoses, Yangzhou, 225009 China; 4 Institute of Agricultural Economics and Information, Henan Academy of Agricultural Sciences, Zhengzhou, 450002 China

**Keywords:** Heat-labile enterotoxin, ETEC, adherence, fimbriae, pathogenesis

## Abstract

As one of the crucial enterotoxins secreted by enterotoxigenic *Escherichia coli* (ETEC), heat-labile enterotoxin (LT) enhances bacterial adherence both in vivo and in vitro; however, the underlying mechanism remains unclear. To address this, we evaluated the adherence of LT-producing and LT-deficient ETEC strains using the IPEC-J2 cell model. The expression levels of inflammatory cytokines and chemokines, and tight-junction proteins were evaluated in IPEC-J2 cells after infection with various ETEC strains. Further, the levels of adhesins and enterotoxins were also evaluated in F4ac-producing ETEC (F4 + ETEC) strains after treatment with cyclic AMP (cAMP). The adherence of the *ΔeltAB* mutant was decreased compared with the wild-type strain, whereas adherence of the 1836-2/pBR322-eltAB strain was markedly increased compared with the 1836-2 parental strain. Production of LT up-regulated the expression of TNF-α, IL-6, CXCL-8, and IL-10 genes. However, it did not appear to affect tight junction protein expression. Importantly, we found that cAMP leads to the upregulation of adhesin production and STb enterotoxin. Moreover, the F4 + ETEC strains treated with cAMP also had greater adhesion to IPEC-J2 cells, and the adherence of *ΔfaeG*, *ΔfliC*, and *ΔestB* mutants was decreased. These results indicate that LT enhances the adherence of F4 + ETEC due primarily to the upregulation of F4 fimbriae, flagellin, and STb enterotoxin expression and provide insights into the pathogenic mechanism of LT and ETEC.

## Introduction

Enterotoxigenic *Escherichia coli* (ETEC) is an important cause of diarrhea and mortality in neonatal and recently weaned pigs [1, 2], causing more than $90 million in losses annually due to the death of up to 5% of young pigs worldwide [2]. ETEC is also the leading cause of diarrhea in children in developing countries (especially those younger than 5 years old) and adults who travel to these areas [3, 4].

The major ETEC virulence factors are adhesins and enterotoxins. In ETEC that causes diarrhea in pigs, the variants of adhesive fimbriae are F4 (K88), F5 (K99), F6 (K99), F18, and F41, in addition to several non-fimbriae adhesins (flagella, AIDA-I) [5–8]. Enterotoxins include heat-labile enterotoxin (LT) and several heat-stable enterotoxins (STp, STb, and EAST1) [9]. Adhesion to epithelial cells is initially mediated by adhesins, which leads to ETEC colonization of the host small intestine. Subsequently, colonized bacteria produce toxins, which results in the loss of electrolytes and culminates in watery diarrhea. Although the mechanism of ETEC disease has been characterized, how the various virulence factors contribute to initial bacterial adherence remains unclear.

As one of the two crucial types of enterotoxins produced by ETEC, LT is a typical A_1_B_5_ toxin [10]. The holotoxin comprises a single LT_A_ subunit and a pentameric LT_B_ subunit [11]. LT shares similar structure, biological function, and pathogenic mechanism with cholera toxin (CT) produced by *Vibrio cholera*, which is responsible for the disease cholera. The LT_A_ subunit exhibits ADP-ribosylation activity and exerts enterotoxicity, whereas the LT_B_ subunit has a membrane-binding function and binds specifically to the GM1 ganglioside receptor on the surface of host cells [12]. Recently, Cervin et al. have discovered that fucosylated glycans expressed in small intestine epithelial cells are the main receptors for CTB, and most likely also for LT [13]. The mechanism by which LT exerts enterotoxicity and induces watery diarrhea is well known. Intriguingly, LT was also found to play an important role in bacterial adherence in vitro and in vivo, beyond its enterotoxicity [14–17].

One of the first pieces of evidence that LT plays a role in enhancing the colonization ability of F4ac + ETEC, which is associated with porcine diarrhea, was observed in a gnotobiotic piglet model [14]. Another study demonstrated that endogenously produced or exogenously added LT increase the adherence of both porcine- and human-derived ETEC strains in vitro [16]. The adherence enhancement is primarily due to the increased expression of F4ac fimbriae, the major adhesin that mediates the initial attachment of F4ac + ETEC to intestinal epithelial cells. A recent study found that LT triggered the production of carcinoembryonic antigen-related cell adhesion molecules (CEACAMs) on human small intestinal epithelial cells, which serve as a receptor for type I fimbriae expressed by human ETEC strains, enhancing bacterial adherence [18]. In addition, it was found that piglets pre-injected with purified LT toxin exhibit enhanced subsequent *Salmonella enterica* infection [17]. LT favors *S. enterica* adherence by decreasing the expression of mucin 4, resulting in disruption of the intestinal mucus layer. An increasing amount of evidence supports a role for LT in mediating bacterial adherence; however, the underlying mechanism by which LT contributes to this process remains poorly understood.

This study aimed to elucidate the exact mechanism by which LT enhances bacterial adherence. Using an IPEC-J2 cell model, we confirmed that LT production promotes F4 + ETEC adhesion to host cells. Moreover, the adherence enhancement is primarily due to the upregulation of bacterial adhesin and STb enterotoxin expression.

## Materials and methods

### Bacterial strains, plasmids, and cell lines

The bacterial strains and plasmids used in this study are listed in Table 1. Wild-type F4 + ETEC strain C83902 (F4^+^, LT^+^, STp^+^, STb^+^) isolated from diarrhea piglet was used for constructing the *ΔeltAB*, *ΔfaeG*, *ΔfliC*, and *ΔestB* mutants [19]. 1836-2 (F4^+^, EAST1^+^) is a nonpathogenic ETEC field isolate used to express LT [15]. The wild-type strains and mutants were grown in LB broth or on LB agar plates. The complemented C83902*ΔeltAB*/pBR322-eltAB and 1836-2/pBR322-eltAB strains were cultivated in LB containing 100 µg/mL ampicillin (Amp+). The porcine small intestinal cell line IPEC-J2 (Bio-106,957) was purchased from Biobw and cultured in Dulbecco’s minimal Eagle medium (Gibco) supplemented with 10% heat-inactivated fetal bovine serum (Gibco).

**Table 1:** **Strains and plasmids used in this study**

Strain/plasmid	Relevant characteristics	Source or reference
DH5α	φ80(lacZ)∆M15 ∆(lacZYA-argF)U169 endA1 recA1 hsdR17(rk^−^, mk^+^)	Tsingke
ETEC C83902	Wild-type K88ac LT^+^, STa^+^, STb^+^	[19]
C83902Δ*eltAB*	Wild-type K88ac LT^−^, STa^+^, STb^+^	This study
C83902Δ*eltAB*/ pBR-eltAB	C83902Δ*eltAB* carrying pBR-eltAB plasmid	This study
C83902Δ*faeG*	C83902 deletion of *faeG* gene	This study
C83902Δ*fliC*	C83902 deletion of *fliC* gene	This study
C83902Δ*estB*	C83902 deletion of *estB* gene	This study
ETEC 1836-2	Wild-type K88ac EAST1^+^	[40]
ETEC 1836-2/pLT	1836-2 carrying pBR-eltAB plasmid	This study
Plasmid		
pBR322-eltAB	pBR322 carrying the full *eltAB* gene	This study
pKD3	Cm^+^; Cm cassette template	[20]
pKD46	Amp^+^, λ-Red recombinase expression	[20]
pCP20	Amp^+^, Cm^+^; Flp recombinase expression	[20]

### **Generation of isogenic mutants for C83902 ETEC and the complemented strain C83902*ΔeltAB*/pBR322-eltAB**

Wild-type ETEC C83902 was chosen as the parent strain to construct the isogenic mutants using the λ-Red recombinant method described previously [20]. Using the *eltAB* gene as an example, the chloramphenicol (Cm)^+^ cassette was amplified from the plasmid pKD3 by PCR using the primers *Δ*LT-F and *Δ*LT-R (Table 2). The purified PCR product was introduced into C83902 competent cells containing plasmid pKD46. The primary recombinant C83902*ΔeltAB::Cat* strain was selected on both Cm^+^ and Amp^+^ LB plates. The Cm-resistance gene was then eliminated by introducing the plasmid pCP20, which produces FLP recombinase at 42 °C. The successful construction of the C83902*ΔeltAB* isogenic mutant was verified by PCR and DNA sequencing combined with GM1-binding and ADP-ribosylation activity assays.

**Table 2:** **Sequences of primer oligonucleotides used in this study**

Name	Primer sequence (5’-3’)
ΔLT-F	ATGAAAAATATAGCTTTCATTTTTTTTATTTTATTAGCATCGCCATTATATGCAAATTGTGTAGGCTGGAGCTGCTTCG
ΔLT-R	TGTTATATAGGTTCCTAGCATTAGACATGCTTTTAAAGCAAACTAGTTTTTCATACTCATATGAATATCCTCCTTA
pBR-LT-F	CGGATTGTCTTCTTGTATGAT
pBR-LT-R	GATCGGTATTGCCTCCTCTAC
Claudin-1-F	ACCCGCACTACGTCACCTTC
Claudin-1-R	GGCAGGACACCTGGTCATTG
Occludin-F	GTGGTAACTTGGAGGCGTCTTC
Occludin-R	CCGTCGTGTAGTCTGTCTCGTA
ZO-1-F	AAGGATGTTTACCGTCGCATT
ZO-1-R	ATTGGACACTGGCTAACTGCT
ZO-2-F	CAGGCGGGAGATGTCAAGT
ZO-2-R	ACCATGACCACCCGATCATTT
ZO-3-F	ATGGCGGTGAGATTCCAGGT
ZO-3-R	TCTGAATGGCAAAGGAGGAGG
β-catenin-F	TGGGACCCTTGTTCAGCTTC
β-catenin-R	AGAGCACAGATGGCAGGTTC
GAPDH(E)-F	CGTTAAAGGCGCTAACTTCG
GAPDH(E)-R	ACGGTGGTCATCAGACCTTC
GAPDH(I)-F	GCACCGTCAAGGCTGAGAAC
GAPDH(I)-R	ATGGTGGTGAAGACGCCAGT
CysG-F	GGCAAGGGACGTTTGAAGAC
CysG-R	CGGCGATGACCAACCAA
HPRT1-F	CGGCTTGCTCGAGATGTGAT
HPRT1-R	GCACACAGAGGGCTACGATGT
TNF-α-F	CGACTCAGTGCCGAGATCAA
TNF-α-R	CCTGCCCAGATTCAGCAAAG
IL-6-F	ATGCTTCCAATCTGGGTTCAA
IL-6-R	CACAAGACCGGTGGTGATTCT
CXCL-8-F	GCTCTCTGTGAGGCTGCAGTT
CXCL-8-R	TTTATGCACTGGCATCGAAGTT
IL-10-F	TGGGTTGCCAAGCCTTGT
IL-10-R	GCCTTCGGCATTACGTCTTC
APN-F	ATCGACAGGACTGAGCTGGT
APN-R	CAAAGCATGGGAAGGATTTC
faeG-F	GGGAGCTGCTTTCGCTTTTT
faeG-R	CCTCGGCAAACCACCATAAA
Flic-F	TGACTCGGGCTGCACGTA
Flic-R	TTCAGTGGTCTGCGCAACAG
STa-F	GCTAATGTTGGCAATTTTTATTTCTG
STa-R	TTTGAAGAGTCAAGTGATTCAGTTGA
STb-F	TGCCTATGCATCTACACAATCAAAT
STb-R	ACCTTTTTTACAACTTTCCTTGGCTAT

The full-length *eltAB* gene was amplified by PCR from ETEC strain C83902 whole-genome DNA using the primers pBR-LT-F and pBR-LT-R (Table 2) and then cloned into the *Eco*RV sites of the pBR322 vector. The recombinant pBR322-eltAB plasmid was then introduced into the isogenic mutant C83902*ΔeltAB* to generate the complemented strain C83902*ΔeltAB*/pBR322-eltAB. The recombinant plasmid was also introduced into wild-type ETEC 1836-2 to construct the strain 1836-2/pBR322-eltAB. Functional expression of LT in strains C83902*ΔeltAB*/pBR322-eltAB and 1836-2/pBR322-eltAB was verified by GM1-binding and ADP-ribosylation activity assays.

### GM1 enzyme-linked immunosorbent assay (ELISA)

GM1 ELISA was performed to assess GM1-binding activity of strains expressing/not expressing LT, as described previously [21]. Briefly, 400 ng/well of GM1 (Sigma, MO, USA) as antigen was coated onto the wells of a 96-well plate (Nest, China). Next, 100 µL of filtered culture medium from C83902, C83902*ΔeltAB*, C83902*ΔeltAB*/pBR322-eltAB, 1836-2, 1836-2/pBR322-eltAB, or DH5α cells was added and incubated at 37 °C for 1 h. After washing with phosphate-buffered saline (PBS) with 0.05% Tween20, each well was incubated with 100 µL of anti-CT antibody (1:1000) and 100 µL of horseradish peroxidase-conjugated goat anti-rabbit IgG (1:5000) at 37 °C for 1 h. The optical density (OD) of each well was then measured at 450 nm 30 min after adding TMB substrate (Beyotime, China) at room temperature.

### cAMP measurement

Filtered culture medium (100 µL) from various strains or LB medium mixed with 400 µL DMEM was added into each well of a 6-well cell culture plate containing a confluent monolayer of IPEC-J2 cells and incubated at 37 °C for 1 h. The culture supernatant was moved to a new tube. The cells were washed three times in cold PBS and 500 µL cell lysis buffer was added to each well to lyse the cells, then centrifuged at 0.1 × *g* for 10 min at 4 °C to remove the cellular debris. Next, cAMP concentration of the culture supernatants and lysate suspension was determined by competitive ELISA using a cAMP Parameter assay kit (R&D Systems, USA) according to the manufacturer’s instructions.

### Bacterial growth curve determination

ETEC C83902, C83902*ΔeltAB*, C83902*ΔeltAB*/pBR322-eltAB, 1836-2, and 1836-2/pBR322-eltAB cells were grown overnight in glass culture tubes at 37 °C on a shaker operated at 200 rpm. The next day, 40 µL of each overnight bacterial culture was sub-cultured in 4 mL of fresh LB medium, and the OD at 600 nm (OD_600_) was measured hourly over a 6-h period.

### Bacterial adherence assays

Bacterial adherence assays were performed as previously described [22]. Briefly, wild-type ETEC strain C83902; mutants C83902*ΔeltAB*, C83902*ΔeltAB*/pBR322-eltAB, C83902*ΔfaeG*, C83902*ΔfliC*, and C83902*ΔestB* and wild-type ETEC strains 1836-2 and 1836-2/pBR322-eltAB were cultured to the logarithmic phase, and then approximately 1 × 10^7^ colony forming units (CFUs) of bacteria were added to a confluent monolayer of IPEC-J2 cells (multiplicity of infection = 30:1) in each well of a 24-well cell culture plate (Nest). After 90 min of co-incubation, non-adherent bacteria were removed by washing with sterile phosphate-buffered saline (PBS), and IPEC-J2 cells with adherent bacteria were dislodged with 1 mL of 0.25% trypsin. The suspensions were mixed, serially diluted (1:10), and plated on LB plates. The number of colonies of adherent bacteria was determined after overnight growth at 37 °C.

The effect of cAMP on ETEC adherence was measured by performing a pre-incubation experiment in which ETEC C83902 and C83902*ΔeltAB* strains were first incubated with 0, 2, 5, or 10 µM cAMP for 3 h at 37 °C. The number of bacteria adhering to the IPEC-J2 cells was determined as described above.

### RNA extraction, reverse transcription, and quantitative real-time PCR (qPCR)

IPEC-J2 cells were co-infected with ETEC C83902, C83902*ΔeltAB*, C83902*ΔeltAB*/pBR322-eltAB, 1836-2, or 1836-2/pBR322-eltAB for 1.5 h at 37 °C and then washed four times with cold PBS. Wild-type ETEC C83902 cells were treated with 0, 2, 5, or 10 µM cAMP for 3 h. Total RNA was extracted from IPEC-J2 cells and various bacterial samples using TRIzol reagent (TIANGEN, China), as previously described [23]. The yield and integrity of RNA were determined by agarose gel electrophoresis. Spectrophotometric RNA quality control was verified using a Nanodrop2000 spectrophotometer (Thermo Scientific, USA) using only samples with a 260/280 ratio between 1.8 and 2.1 and 260/230 ratio between 1.5 and 2.0.

Total RNA (2 µg) of each sample was used to synthesize the first-strand complementary DNA by a FastKing gDNA Dispelling RT SuperMix Kit (TIANGEN, China) according to the manufacturer’s instructions. The endogenous reference genes glyceraldehyde-3-phosphate dehydrogenase (*GAPDH*) and Hypoxanthine-guanine phosphoribosyltransferase1 (*HPRT1*) were chosen as reference genes for *E. coli*, while *GAPDH*(*I*) and *cysG* genes were chosen as the reference genes for IPEC-J2. Primers for *GAPDH*(*E*), *cysG*, *GAPDH*(*I*), *HPRT1*, *Claudin-1*, *Occludin*, *ZO-1*, *ZO-2*, *ZO-3*, *β-catenin*, *TNF-α*, *IL-6*, *CXCL-8*, *IL-10*, APN, *STp*, *STb*, *faeG*, *etpA*, and *fliC* were designed using the Primer Express 3.0.1 software (Thermo Fisher Scientific, USA). Primer concentrations were measured during optimization reactions using pooled cDNA. qPCR amplification was carried out using AceQ qPCR SYBR Green Master Mix (Vazyme, China) according to the manufacturer’s instructions. For each sample, 100 ng of cDNA and 300 nM concentrations of each primer set were mixed with 10 µL of AceQ qPCR SYBR Green Master Mix per well. A two-step program was run in the 7500 Real-time system (Applied Biosystems, USA). Thermal cycling conditions were 95 °C for 5 min, followed by 40 cycles of 95 °C for 10 s and 60 °C for 30 s. Melting-curve analysis confirmed primer was gene-specific and not due to primer dimers. All reactions were run in quadruplicates. The data from bacteria were normalized to the two reference genes *GAPDH*(*E*) and *cysG*, while data from IPEC-J2 were normalized to the reference genes *GAPDH*(*I*) and *HPRT1*. The results were analyzed using the 2^−ΔΔCT^ method. All primers used for qPCR assays are indicated in Table 2.

### Western blotting

F4 fimbriae and flagellar expression were detected by western blot analysis using whole-cell lysates. Briefly, ETEC C83902 bacteria were pre-treated with 0, 2, 5, or 10 µM cAMP for 3 h; the bacterial culture was then adjusted to the same OD_600_ value (OD_600_ = 1.0). 1mL of each of the above bacterial cultures was collected and re-suspended by 80 µL PBS and 20 µL 5×SDS loading buffer. The whole bacteria were lysed by boiling for 10 min in the loading buffer. Then, 20 µL of whole-cell lysates from the above bacterial cultures were electrophoresed on a 12% denaturing sodium-dodecyl-sulfate-polyacrylamide gel (SDS-PAGE), then transferred onto a nitrocellulose membrane filter. Western blot was performed with mouse anti-FaeG serum (1:15 000) or rabbit anti-fliC_H19_ (1:20 000) serum as primary antibodies and HRP-labeled goat anti-mouse/rabbit immunoglobulin IgG (1:10 000, Abclonal, China) as secondary antibodies. An enhanced chemiluminescent (ECL) kit (NCMBiotech, Suzhou, China) was used to detect the corresponding bands.

### Statistical analysis

All statistical analysis was performed with GraphPad Prism 7.0 software. Data are expressed as the mean with the standard deviation (SD). Student’s *t*-test statistical analysis was used for statistical comparisons of differences between two groups. The difference in more than two groups was analyzed by one-way ANOVA, followed by a Dunnett’s multiple comparison post hoc test. The symbol * denotes *p* < 0.05, indicating a significant difference between the groups, and ** denotes *p* < 0.01, indicating a highly significant difference between the groups.

## Results

### **Characterization of the C83902*ΔeltAB*isogenic mutant and 1836-2/pBR322-eltAB strains**

**Figure 1:**
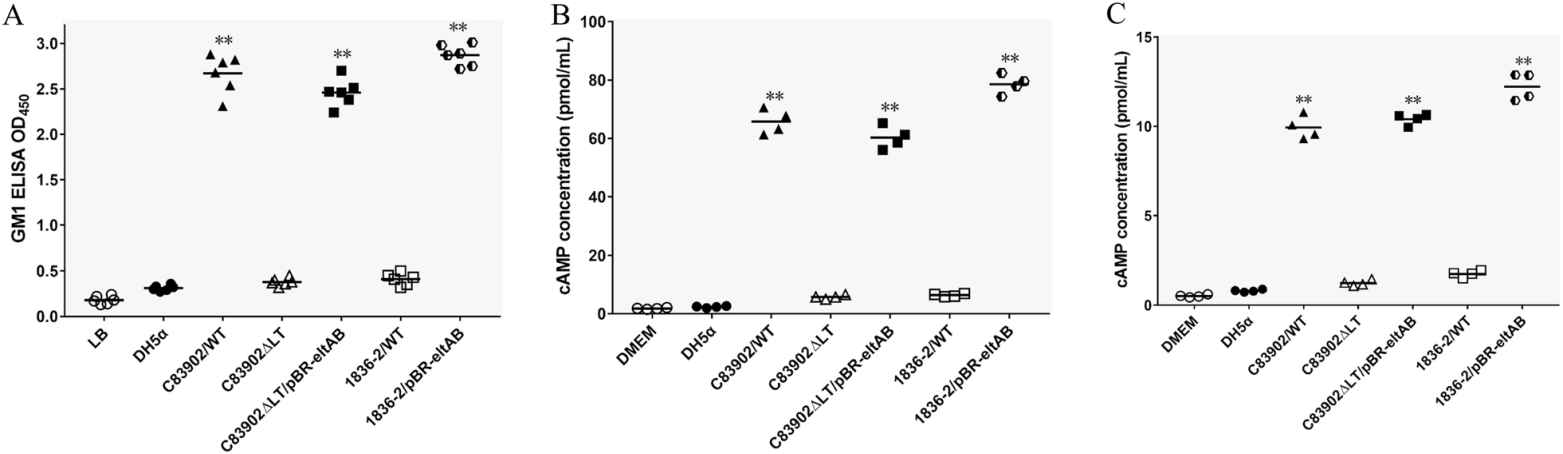
**Characterization of LT-expressing and -deficient strains. A** GM1-binding ability of filtered culture medium from wild-type ETEC strains C83902 and 1836-2, C83902*ΔeltAB* mutant, C83902*ΔeltAB*/pBR322-eltAB complemented, and 1836-2/pBR322-eltAB strains. The mean from six independent experiments is presented. **B** cAMP levels in lysates of IPEC-J2 cells incubated with the indicated bacterial strains. The mean from four independent experiments is presented. **C** cAMP levels in culture supernatants of IPEC-J2 cells incubated with the indicated bacterial strains. The mean from four independent experiments is presented. WT, wild-type.

ETEC strain C83902, which expresses the F4ac fimbriae and LT enterotoxin, is a wild-type strain isolated from a pig with watery diarrhea. This strain was chosen as the parent strain to construct the *ΔeltAB* isogenic mutant. The filtered culture medium from LT-deficient C83902*ΔeltAB* mutant has lost the ability to bind GM1 receptors (Figure 1A). Compared to those infected with the wild-type C83902 strain, the cAMP levels in IPEC-J2 cell lysates (Figure 1B) and culture supernatants (Figure 1C) of cells infected with the C83902*ΔeltAB* mutant were significantly reduced. Culture medium from the complemented C83902*ΔeltAB*/pBR322-eltAB strain restored its ability to bind GM1 receptors. cAMP levels from IPEC-J2 cells infected with the complemented strain were similar to those of the cells infected with the parent strain. Unlike the 1836-2 parent strain, the 1836-2/pBR322-eltAB strain harboring the pBR322-eltAB plasmid strongly bound GM1 (Figure 1A) and elicited high cAMP production (Figures 1B and C). cAMP concentration in IPEC-J2 cell lysates and culture supernatants infected with the 1836-2/pBR322-eltAB strain was significantly higher than cAMP concentration of cells stimulated by the wild-type 1836-2 strain. Moreover, all strains exhibited similar growth rates, indicating that LT expression does not influence bacterial growth.

#### LT promotes F4 + ETEC adhesion to porcine epithelial cells

**Figure 2:**
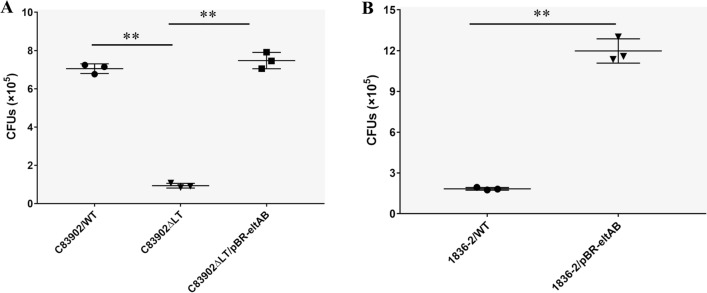
**Production of LT enhances F4 + ETEC adhesion to IPEC-J2 cells**. **A** Adhesion to IPEC-J2 cells by WT ETEC C83902, C83902*ΔeltAB* mutant, and complemented C83902*ΔeltAB*/pBR322-eltAB strains. **B **Adherence to IPEC-J2 cells by WT ETEC1836-2 and 1836-2/pBR322-eltAB strains. Data are expressed as mean ± SD of triplicate experiments.

To examine the effect of LT on the adherence of F4ac + ETEC strains, the adherence capability of wild-type ETEC C83902, the C83902*ΔeltAB* mutant deficient in LT expression, the complemented strain C83902*ΔeltAB*/pBR322-eltAB, wild-type ETEC 1836-2, and 1836-2/pBR322-eltAB expressing LT in the 1836-2 strain was examined using an IPEC-J2 cell model. As shown in Figure 2A, the C83902*ΔeltAB* mutant exhibited a significant reduction in the ability to adhere to IPEC-J2 cells compared with the parent strain (*p* < 0.01), whereas the complemented strain exhibited adherence ability similar to that of the wild-type strain. Bacterial CFU quantification showed that the *ΔeltAB* mutant exhibited approximately 87% less adherence than the parental C83902 strain. Moreover, expression of LT in the 1836-2 strain (1836-2/pBR322-eltAB) resulted in highly enhanced adherence compared with the wild-type 1836-2 strain (Figure 2B, *p* < 0.01). The adherence ability of the 1836-2/pBR322-eltAB strain was approximately 6.5-fold greater than that of the wild-type 1836-2 strain. These results indicate that LT promotes F4 + ETEC bacterial adherence.

### LT expression increases inflammatory cytokines and chemokines expression in IPEC-J2 cells

**Figure 3:**
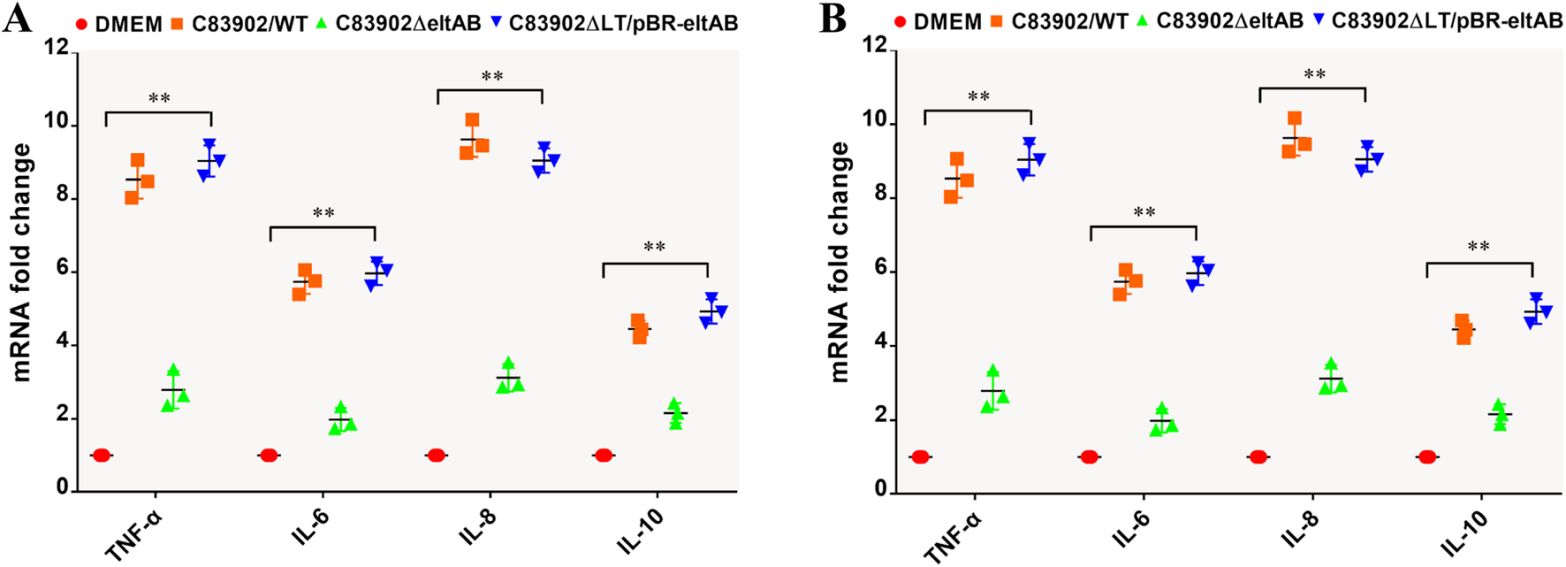
**LT expression results in increased production of inflammatory cytokines and chemokines in IPEC-J2 cells**.** A **Change in the mRNA expression levels of TNF-α, IL-6, IL-8, and IL-10 following infection with WT ETEC C83902, C83902*ΔeltAB* mutant, and complemented C83902*ΔeltAB*/pBR322-eltAB strains. **B** Change in the mRNA expression levels of TNF-α, IL-6, IL-8, and IL-10 following infection with WT ETEC1836-2 and 1836-2/pBR322-eltAB strains. The data are expressed as the mean ± SD of triplicate experiments.

To determine the role of LT in stimulating inflammatory cytokines and chemokines production, the transcript expression levels of TNF-α, IL-6, CXCL-8, and IL-10 were measured by RT-qPCR. The results showed that the production of these cytokines and chemokines by IPEC-J2 cells infected with the *ΔeltAB* mutant was significantly reduced compared with cells infected with the wild-type strain (Figure 3A, *p* < 0.01). Deletion of the *eltAB* gene decreased the expression of the TNF-α, IL-6, CXCL-8, and IL-10 genes by 4.87 ± 0.36-, 5.79 ± 0.28-, 4.53 ± 0.46-, and 3.97 ± 0.27-fold, respectively. Infection with the complemented C83902*ΔeltAB*/pBR322-eltAB strain restored the expression of these inflammatory cytokine and chemokine genes to the levels induced by wild-type infection. The expression levels of the TNF-α, IL-6, CXCL-8, and IL-10 genes by cells infected with 1836-2/pBR322-eltAB were significantly higher than those induced by the parental 1836-2 strain (Figure 3B, *p* < 0.01). These data suggest that higher level of inflammatory cytokines and chemokines production of IPEC-J2 cells infected with LT-producing ETEC strains is mainly attributed to the secretion of LT.

### Expression of tight junction proteins is not altered by LT

To evaluate the effect of LT on the intestinal epithelial barrier, the expression of tight junction proteins was examined in IPEC-J2 cells using RT-qPCR. The data showed that IPEC-J2 cells infected with the *ΔeltAB* mutant did not exhibit any significant change in the expression of claudin-1, occludin, ZO-1, ZO-2, ZO-3, or β-catenin compared with cells infected with the parental strain. Similarly, there was no significant difference in the levels of these junction proteins between IPEC-J2 cells infected with wild-type 1836-2 and the 1836-2/pBR322-eltAB strains. These results indicate that LT does not appear to affect the expression of tight junction proteins in IPEC-J2 cells.

### cAMP enhances adhesins expression by F4 + ETEC strains

**Figure 4:**
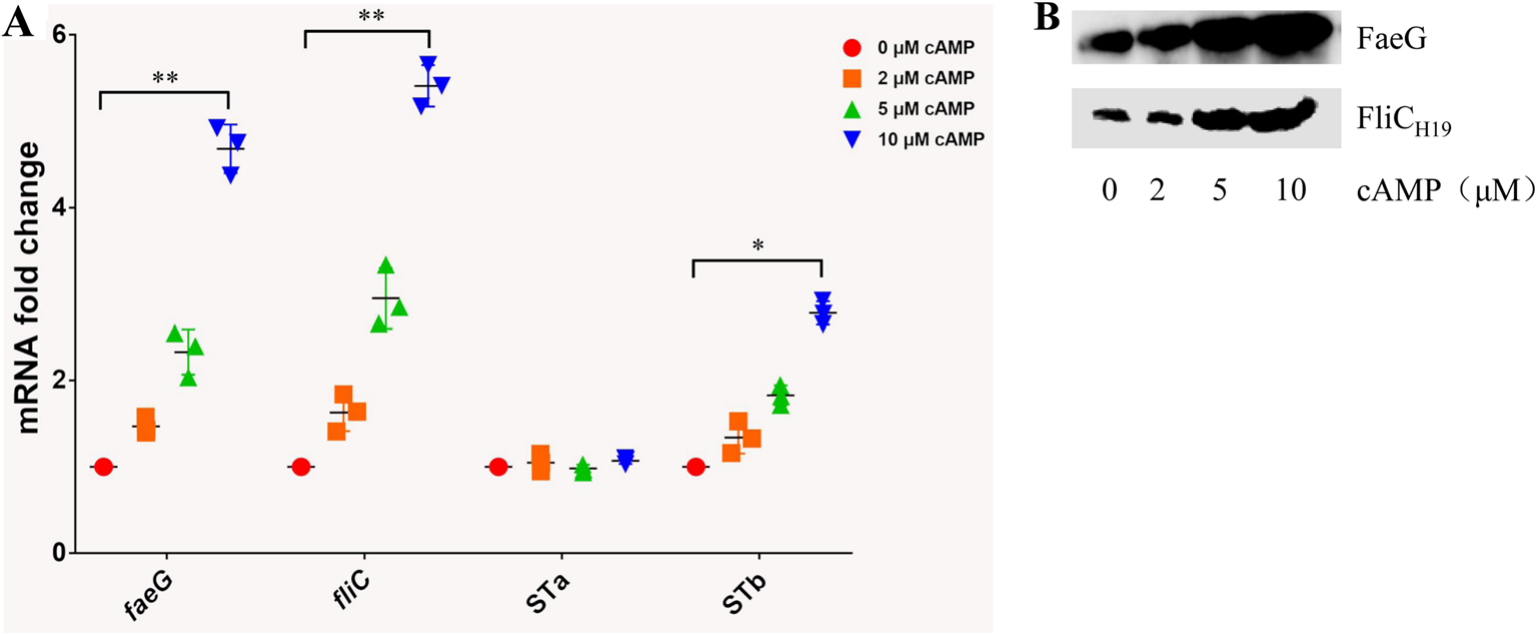
**Treatment with cAMP up-regulates the expression of F4 fimbriae, flagellin, and STb**. **A**Wild-type ETEC C83902 was incubated with 0, 2, 5, or 10 µM cAMP for 3 h, with mRNA expression of *faeG*, *fliC*, *estA* (STp), and *estB* (STb) was examined by qRT-PCR. The mRNA level of *faeG*, *fliC*, *estA*, and *estB* genes without cAMP treatment was considered 100%. The data are expressed as the mean ± SD of four experiments. **B** The protein expression of F4 fimbriae and flagellin from ETEC C83902 after treatment of with 0, 2, 5, or 10 µM cAMP.

Next, we treated the wild-type C83902 strain with various concentrations of cAMP (0–10 µM) to evaluate potential changes in bacterial adhesins expression. Expression of FaeG and FliC was highly increased at both transcript level (Figure 4A) and protein level (Figure 4B). As shown in Figure 4A, Expression of FaeG and FliC was up-regulated 2.33 ± 0.12- and 2.95 ± 0.12-fold, respectively, in cells treated with 5 µM cAMP and 4.68 ± 0.18- and 5.51 ± 0.23-fold, respectively, in cells treated with 10 µM cAMP. These results indicate that cAMP increases F4 fimbriae and flagellin expression in a concentration-dependent manner.

### cAMP enhances STb toxin expression

To explore whether cAMP stimulated by LT affects the expression of other enterotoxins in F4 + ETEC, the mRNA levels of *estA* (STp) an*d estB* (STb) were examined under stimulation with different concentrations of cAMP. The results showed that cAMP profoundly enhanced STb enterotoxin expression (Figure 4A). The expression of STb enterotoxin was up-regulated approximately 1.8- and 2.8-fold in cells treated with 5 and 10 µM cAMP, respectively. However, the expression of STp enterotoxin remained unchanged under each treatment condition (Figure 4A).

### cAMP treatment promotes F4 + ETEC adherence

**Figure 5:**
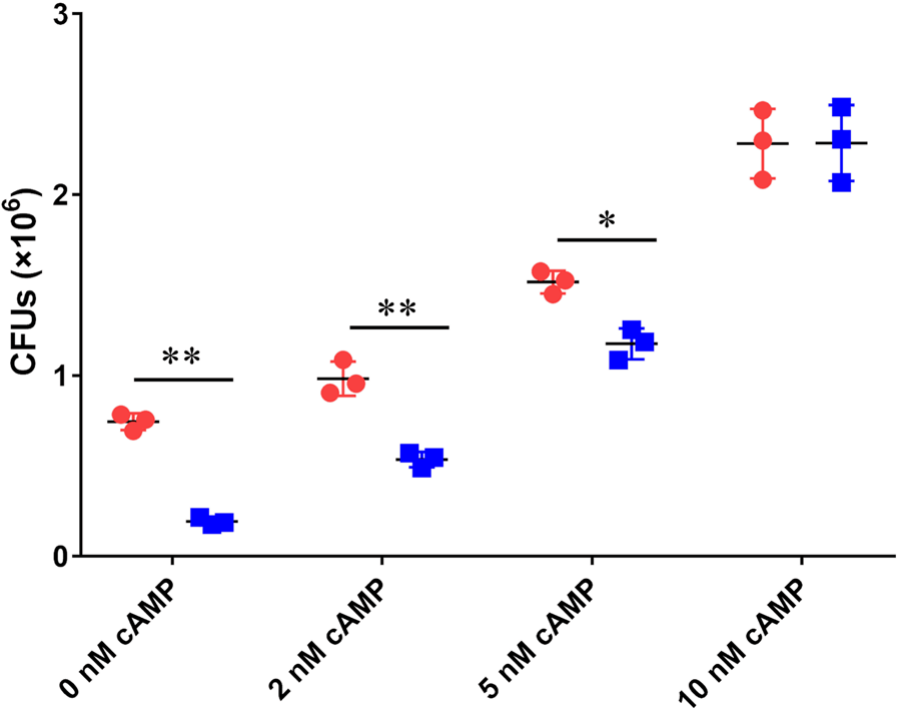
**Treatment with cAMP increases the adhesion of F4 + ETEC to IPEC-J2 cells.** WT ETEC C83902 and C83902*ΔeltAB* mutant strains were cultured to the logarithmic phase in the presence of 0, 2, 5, or 10 µM cAMP. The MOI of 30 bacteria per cell was added to each well of a 24-well cell culture plate and incubated in a CO_2_ incubator for 1.5 h. The number of colonies of adherent bacteria was determined by serial dilution (1:10). The data are expressed as the mean ± SD of triplicate experiments.

To determine if the increased expression of F4 fimbriae, flagellin, and STb enterotoxin induced by cAMP may be responsible for increased ETEC adherence, we compared the ability of wild-type C83902 and C83902*ΔeltAB* mutant to adhere to IPEC-J2 cells either treated with cAMP or not. The results showed that cAMP treatment led to a significant increase in wild-type C83902 and C83902*ΔeltAB* mutant adherence. The adherence ability of wild-type C83902 was increased about 1.3-, 2.0-, and 3.0-fold after being treated with 2, 5, and 10 µM cAMP, respectively (Figure 5). Meanwhile, the adherence ability of the *ΔeltAB* mutant was increased about 2.7-, 6.0-, and 12.0-fold after being treated with 2, 5, and 10 µM cAMP, respectively (Figure 5). These results suggest that cAMP enhances ETEC adherence and may be mediated by the increased F4 fimbriae, flagellin, and STb enterotoxin expression.

### ***ΔfaeG***, ***ΔfliC***, **and*ΔestB*isogenic mutants decrease adhesion to IPEC-J2 cells**

**Figure 6:**
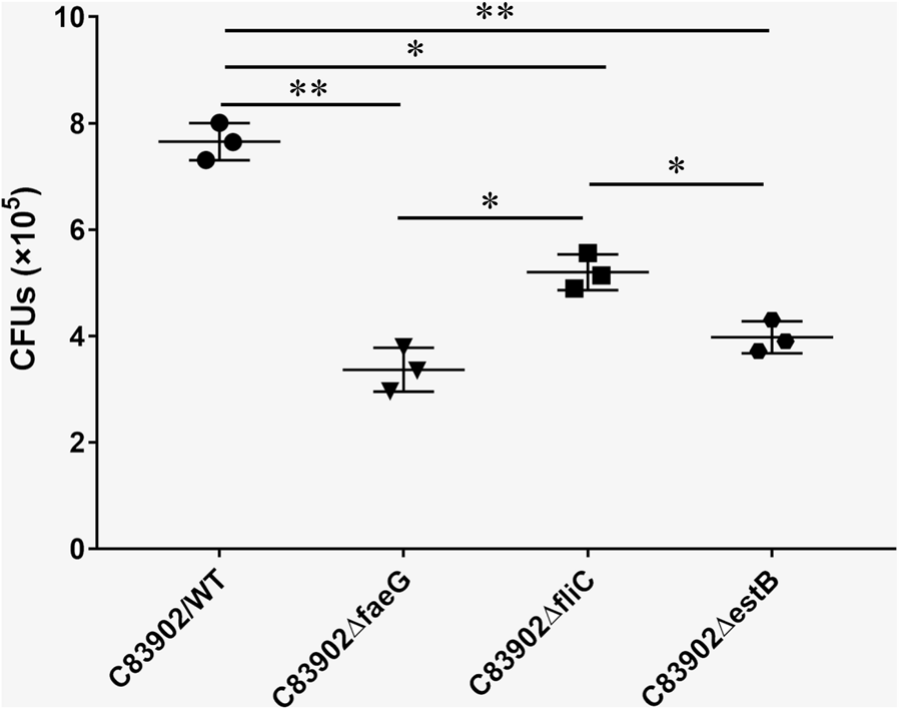
***ΔfaeG***, ***ΔfliC***, **and** ***ΔestB*** **mutants decrease the adhesion to IPEC-J2 cells.** Quantification of WT ETEC C83902 and its *ΔfaeG*, *ΔfliC*, and *ΔestB* mutants adhesion to porcine IPEC-J2 cells. The data are expressed as the mean ± SD of triplicate experiments

As shown in Figure 6, C83902*ΔfaeG*, C83902*ΔfliC*, and C83902*ΔestB* mutants significantly reduced the ability to adhere to IPEC-J2 cells relative to the parent strain. Quantification of bacterial colony-forming units (CFUs) showed that the C83902*ΔfaeG* isogenic mutant had an 56% decrease in adherence compared to the wild-type C83902 strain. The C83902*ΔfliC* mutant showed a 32% reduction in its ability to adhere to IPEC-J2 cells. While the C83902*ΔestB* mutant also exhibited 48% less adherence than the parent strain. This result indicates that F4 fimbriae, flagella, and STb enterotoxin are involved in F4 + ETEC adhesion to IPEC-J2 cells.

## Discussion

The mechanisms by which LT promotes enteric pathogens adherence remain unclear [14, 16, 24, 25]. To investigate the role of LT in promoting ETEC adherence, we evaluated the adherence ability of an ETEC wild-type strain C83902 (LT+) ETEC that was isolated from diarrhea piglet and C83902*ΔeltAB* mutant (LT-), as well as a non-LT producing 1836-2 ETEC strain and its LT-expressing strain 1836-2/pBR322-eltAB using IPEC-J2 cell model. IPEC-J2 is a non-transformed columnar epithelial cell line that was isolated from neonatal piglet mid-jejunum. IPEC-J2 cells are morphologically and functionally similar to intestinal epithelial cells with mucins and tight junctions, they produce cytokines and chemokines and express toll-like receptors. This cell line is thus an ideal in vitro model for studying the pathogenesis of enteric pathogens, complex pathogens-host interactions, and host immune responses [16, 19, 24, 26, 27]. Consistent with previous results, we found that the adherence ability of the *ΔeltAB* mutant was dramatically impaired compared with the wild-type strain, whereas the adherence ability was restored in the complemented strain [16, 24]. Moreover, expressing LT in the ETEC 1836-2 strain markedly increased the adhesion to IPEC-J2 cells. Our data confirmed the role of LT in promoting ETEC adherence in vitro. Furthermore, we found that LT enhanced ETEC adherence by increasing F4 fimbriae, flagella, and STb enterotoxin expression.

This study makes four important novel contributions. First, our results demonstrate that LT enhances F4 + ETEC adhesion to IPEC-J2 not only by increasing the expression of F4 fimbriae (mRNA and protein level) but also flagella (mRNA and protein level) and STb (mRNA level). Furthermore, these results were confirmed by showing that the adherence of the C83902*ΔfaeG*, C83902*ΔfliC*, and C83902*ΔestB* mutants was significantly decreased compared to the parent strain. Second, we tested the pro-inflammatory cytokines and chemokines, and tight junction gene expression of IPEC-J2 after being infected with LT-production or LT-deficient strains. Our results show that LT production increases inflammatory cytokine expression (TNF-α, IL-6, IL-8, and IL-10). Though the tight junction of cells is not altered, we cannot exclude that the increased inflammatory factors elevated by LT may impair cell structure in other ways (apoptosis or pyroptosis) to promote adhesion. Third, we treated the strains with cAMP, not the LT-treated IPEC-J2 culture supernatants. In fact, the components in the cell supernatant are too complex to completely rule out the influence of other factors on F4 fimbriae expression. Adding purified cAMP into in vitro bacterial cultures can more directly evaluate cAMP effect (induced by LT enterotoxin) on adhesins expression. Fourth, the infection caused by pathogens is multifactorial, which is considerably more complex than previously known. Our results in this study demonstrate that LT, STb, F4, fimbriae, and flagella are crosstalk in F4 + ETEC.

Intestinal epithelial cells are important parts of activating innate immunity to infection caused by enteric pathogens. A previous study has demonstrated that porcine IEC infection of F4 + ETEC could induce pro-inflammatory cytokines production, and F4 fimbriae and flagellin play a crucial role in this process [28]. Consistent with this study, we determined the relative gene expression of inflammatory cytokines TNF-α, IL-6, CXCL-8, and IL-10 in IPEC-J2 cells after being infected with LT-producing or LT-deficient F4 + ETEC, which indicates the regulation of inflammatory responses upon stimulation of LT in vitro. Although IPEC-J2 cells more closely mimic the intestinal system than other cell lines, it cannot represent the complex physiologically intestinal system due to that it is just a homogenous cell population. It has been reported that several immune-related molecules are undetectable or expressed at very low levels in this cell line [28, 29]. In addition, IPEC-J2 is a non-transformed columnar epithelial cell line. To mimic in vivo conditions, it must be cultured on Transwell membrane inserts to form polarized functional monolayers and tight junctions in vitro. Therefore, our obtained results need to be further confirmed at the protein level and in the differentiated cells.

Inflammatory processes are important for tissue repair and the elimination of pathogens. However, excessive inflammation disrupts epithelial tight junctions and promotes tissue damage. The intestinal epithelium functions as a barrier in the host first-line defense against ETEC infection, and tight junction proteins play an important role in maintaining the intestinal mucosal barrier function [30–32]. Pro-inflammatory cytokines secreted in response to enteric pathogens may also disrupt epithelial tight junctions and contribute to increased bacterial adherence. Therefore, we further study whether the enhanced ETEC adherence disrupts the tight junction of IPEC-J2 cells induced by LT-stimulating an increased inflammatory cytokines and chemokines production. However, a previous study reported that ETEC infection would disrupt the tight junctions structures of the mucosal barrier by reducing the expression of tight junction proteins [33]. However, LT secretion does not appear to affect the expression of tight junction proteins, suggesting that LT-enhanced bacterial adherence is not achieved by impairing the intestinal mucosal barrier.

We then examined the expression of F4 fimbriae receptors, which might also contribute to increased adherence. High-molecular-weight intestinal mucin-type sialoglycoproteins (IMTGPs) and porcine aminopeptidase N (APN) are two receptors for F4 fimbriae thus far identified [34–36]. A previous study demonstrated that LT does not affect IMTGP receptor expression [16]. Melkebeek et al. have demonstrated porcine APN is an endocytotic receptor for F4 fimbriae [36]. In this study, we demonstrate that the production of LT also does not affect the expression of the APN receptor (data not shown). These results suggest that the increased bacterial adherence induced by LT is not due to the upregulation of F4 fimbriae receptor expression. Recently, Sheikh et al. demonstrated that LT increased type I fimbriae receptor expression and subsequently enhanced human ETEC strains adhesion to human small intestinal epithelial cells [18]. Thus, we cannot rule out the possibility that LT increases the expression of other fimbriae receptors to facilitate F4 + ETEC adhesion to IPEC-J2 cells.

cAMP is a critical cellular “second messenger” that plays a role in regulating bacterial virulence determinants. Adhesins are required for initial pathogen-host interactions and play an important role in ETEC pathogenesis. Thus, we hypothesized that the increased adherence associated with LT expression might be attributed to enhanced bacterial adhesins expression. F4 fimbriae and flagella are two known major adhesins that mediate the adhesion of F4 + ETEC to host cells [19, 37]. Previous studies demonstrated that cAMP stimulation was able to increase the expression of F4, 987P, and type I fimbriae [16, 38, 39]. Consistent with previous results, our results revealed that pretreatment of wild-type C83902 and C83902*ΔeltAB* mutant with cAMP can also significantly increase their F4 fimbriae and flagella expression, and subsequent adhesion to IPEC-J2 cells. However, the cAMP concentrations used for stimulating of F4 + ETEC strain in this study are higher than the physiologically relevant ones. Thus, further research is needed to confirm whether physiological concentrations of cAMP have the same effect on enhancing F4 + ETEC virulence factors expression and adhesion ability.

Virulence factors contribute to pathogen infection in multiple ways. In addition to fluid accumulation, LT enterotoxin enhances bacterial adherence both in vitro and in vivo. In contrast to the findings of Erume et al. [40], we observed that the elevated STb enterotoxin by cAMP increases F4 + ETEC adherence to IPEC-J2 cells, while deletion of STb leads to reduce in adherence. This might be explained by the in vitro cell models lacking the fluid flushing effect that occurs in vivo. Previous studies have shown that STb enterotoxin can alter mucosal tight junction protein expression [41] and induce cell apoptosis [42]. Therefore, STb enterotoxin increased bacterial adherence may also be achieved by disrupting the integrity of the intestinal barrier. It has been reported that the expression of LT, STp, and STb enterotoxins are co-regulated in an F4ac + ETEC strain that produced the three enterotoxins [23]. Recently, it was reported that heat sensitive molecules secreted by IPEC-J2 cells increased the STp and STB enterotoxins secretion by ETEC [43]. However, the precise mechanism by which the production of LT co-regulates the expression of other virulence factors is unclear and needs further study.

In summary, the present results indicate that LT can up-regulate F4 fimbriae, flagella, and STb enterotoxin expression. This may partly contribute to LT promoting ETEC adhesion to intestinal epithelial cells. Moreover, the expression of LT and other virulence factors is co-regulated in ETEC. Our results suggest that LT contributes to ETEC pathogenesis in several ways. These findings provide insights into the functions and pathogenic mechanisms of LT and could facilitate the development of new strategies for preventing ETEC infection.

## Abbreviations

APN: Aminopeptidase N
cAMP: Cyclic AMP
CEACAMs: Carcinoembryonic antigen-related cell adhesion molecules
CFUs: Colony-Forming Units
CT: Cholera toxin
ELISA: enzyme-linked immunosorbent assay
ETEC: enterotoxigenic Escherichia coli
IMTGPs: High-molecular-weight intestinal mucin-type sialoglycoproteins
LT: Heat-labile enterotoxin
OD: Optical density
PBS: Phosphate-buffered saline
qPCR: Quantitative real-time PCR
SDS-PAGE: Sodium-dodecyl-sulfate-polyacrylamide gel
SD: Standard deviation
